# Chemotherapy and Anesthesia in Colorectal Cancer Survivors and the Risk of Alzheimer’s Disease

**DOI:** 10.1093/geroni/igaa057.845

**Published:** 2020-12-16

**Authors:** Igor Akushevich, Arseniy Yashkin, Julia Kravchenko, Miklos Kertai

**Affiliations:** 1 Duke University, Durham, North Carolina, United States; 2 Vanderbilt University Medical Center, Nashville, Tennessee, United States

## Abstract

Exposures common in cancer patients––chemotherapy, surgical injury and/or anesthesia, alone or in combination with predisposing factors––have been suggested as potential risk factors for Alzheimer’s disease (AD). We explored the relationship between chemotherapy and cumulative anesthesia exposure, and development of AD in colorectal cancer survivors. We conducted a retrospective cohort study of individuals age 65 and older diagnosed with colorectal cancer between 1998 and 2013, drawing on SEER-Medicare data and employing a proportional hazards model. We found that exposure to chemotherapy in colorectal cancer survivors demonstrated a protective effect for AD HR=0.821 (0.784-0.860). The beneficial effect held in race-, sex-, cancer-stage-specific subgroups, across chemotherapy agents (e.g., Fluorouracil, Oxaliplatin, or Fluorouracil+Leucovorin), in multivariable analyses, and in propensity score-based pseudorandomization based on 70 demographic, socioeconomic, cancer-diagnosis-related, and comorbidity variables. The effect was diminished or absent when non-AD dementias were analyzed. Findings further demonstrated that the association between chemotherapy exposure and AD was not affected by competing risk of long-term mortality or possible correlation between choosing chemotherapy and higher cognitive score or use of alternative health insurance. The effect of anesthesia on AD was not significant (0.998 per hour, 0.992-1.005) and this effect held in all subgroups, multivariable analyses, and for pseudorandomized subpopulations. Harmful effect was detected for cerebral degeneration, excluding AD, cognitive deficits following cerebral hemorrhage, cognitive disorder due to injury, hepatic encephalopathy, and hepatolenticular degeneration. Sensitivity analyses focused on SEER-Registry-specific effects and possible misspecifications in anesthesia records with alternative models demonstrated stability of estimates.

